# Adjunctive medical expulsive therapy with tamsulosin for repeated extracorporeal shock wave lithotripsy: a systematic review and meta-analysis

**DOI:** 10.1590/S1677-5538.IBJU.2020.0093

**Published:** 2020-11-18

**Authors:** Wei Ouyang, Guoliang Sun, Gongwei Long, Man Liu, Hua Xu, Zhiqiang Chen, Zhangqun Ye, Heng Li, Yucong Zhang

**Affiliations:** 1 Huazhong University of Science and Technology Tongji Medical College Hubei Institute of Urology Wuhan China Hubei Institute of Urology, Tongji Hospital, Tongji Medical College, Huazhong University of Science and Technology, Wuhan, China;; 2 Huazhong University of Science and Technology Tongji Medical College Department of Urology Wuhan China Department of Urology, Tongji Hospital, Tongji Medical College, Huazhong University of Science and Technology, Wuhan 430030, China;; 3 Huazhong University of Science and Technology Tongji Medical College Department of Geriatrics Wuhan China Department of Geriatrics, Tongji Hospital, Tongji Medical College, Huazhong University of Science and Technology, Wuhan 430030, China

**Keywords:** Urolithiasis, Tamsulosin, Lithotripsy

## Abstract

**Purpose:**

To evaluate the efficacy of adjunctive medical expulsive therapy (MET) with tamsulosin for the promotion of stone fragments clearance for repeated extracorporeal shock wave lithotripsy (ESWL).

**Materials and Methods:**

This meta-analysis was conducted by systematic search for randomized controlled trial (RCT) studies in PubMed/Medline, Scopus, Cochrane Library, Web of Science databases in January 2020, which compared tamsulosin with either placebo or non-placebo control for repeated ESWL. The primary endpoint was stone-free rate (SFR), the second endpoints were stone clearance time and complications. The quality assessment of included studies was performed by using the Cochrane System and Jadad score.

**Results:**

7 RCTs were included in this meta-analysis. Tamsulosin provided higher SFR (for stones larger than 1cm, OR: 5.56, p=0.0003), except for patients with stones less than 1cm. For patients with renal stones (OR: 2.97, p=0.0005) or upper ureteral stones (OR: 3.10, p=0.004), tamsulosin can also provide a higher SFR. In addition, tamsulosin provided a shorter stone clearance time (WMD: −9.40, p=0.03) and lower pain intensity (WMD=-17.01, p <0.0001) and incidences of steinstrasse (OR: 0.37, p=0.0002).

**Conclusion:**

Adjunctive MET with tamsulosin is effective in patients with specific stone size or location that received repeated ESWL. However, no well-designed RCT that used computed tomography for the detection and assessment of residual stone fragments was found. More studies with high quality and the comparison between tamsulosin and secondary ESWL are needed in the future.

## INTRODUCTION

Urolithiasis is a very common disease in the World with prevalence rates varying from 1% to 20% (1). Though much progress has been made in endourological technology, for patients with kidney and upper ureteral stones, extracorporeal shock wave lithotripsy (ESWL) is still considered to be the initial treatment after its introduction in the early 1980s (2).

Unfortunately, the success of ESWL is not satisfactory enough. It depends on the types of lithotripter, stones characteristics and geographic regions (3). Residual stone fragments may lead to some significant problems to the patient such as colic pain or reintervention. Medical expulsive therapy (MET) was used for promoting the spontaneous passage of stone fragment after ESWL and reducing the stone expulsion time and analgesic requirements (4-6). Nowadays, tamsulosin is the most common agents used in adjunctive MET after ESWL with large amount of relevant published studies (7). However, some randomized controlled trials (RCTs) showed conflicting results, especially for patients received ESWL for more than once. We conducted this systematic review and meta-analysis of evidence from RCTs to evaluate the efficacy of adjunctive MET with tamsulosin for repeated ESWL, primarily in the terms of stone-free rates (SFR), stone clearance time and complications.

## MATERIALS AND METHODS

### Data sources and literature search

**Supplementary Figure 1:**
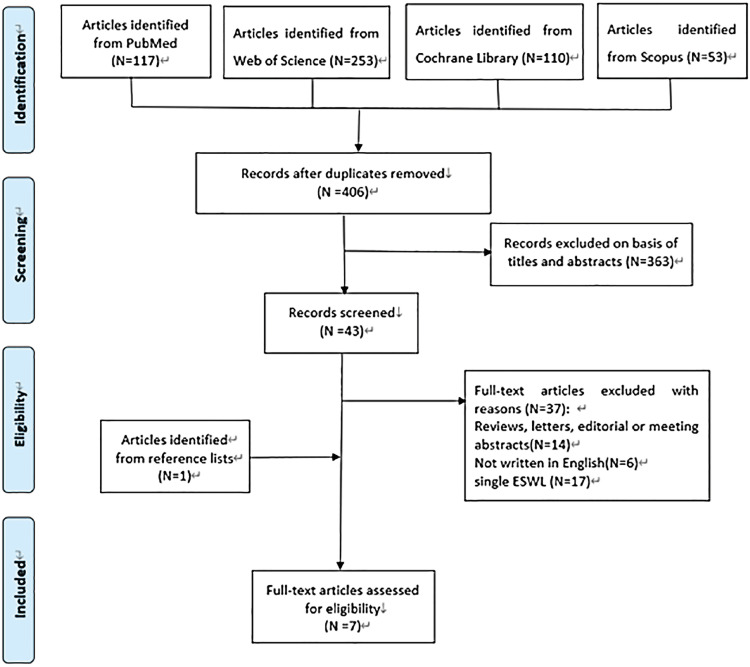
Flow diagram according to preferred reporting items for systematic reviews and meta-analysis

This meta-analysis was conducted through comprehensive research of PubMed/Medline, Scopus, Cochrane Library, Web of Science databases with the search terms of “(medical expulsive therapy OR tamsulosin) AND (extracorporeal shock wave lithotripsy OR shock wave lithotripsy OR ESWL OR SWL) AND (urolithiasis OR calculi OR nephrolithiasis OR kidney stone OR ureter stone)” before January 2020 according to the Preferred Reporting Items for Systematic Review and Meta-Analysis (PRISMA) statement (8). The search flow diagram is presented in Supplementary Figure-1. Only literatures reporting results of RCTs about comparison between tamsulosin and placebo control were included for further screening. Cited references of selected articles were also screened. Literatures without full text were excluded. Two reviewers screened all studies according to inclusion and exclusion criteria independently. Any disagreements were resolved by discussion, and unsolved disagreement was dealt by the third author.

The inclusion criteria for the studies were as follows: 1) enrolling patients with stones received ESWL for more than once; 2) enrolling patients with stones received tamsulosin for ESWL; 3) reporting original research; 4) adult studies; 5) studies written in English. Reviews, studies with a sample size <10 were excluded.

### Data abstraction

Two reviewers manually extracted data from included study using a standardized form independently. Baseline characteristics of these studies were abstracted. Parameters below were assessed in this study: SFR, stone-clearance time, complications and adverse reactions. Pain intensity was assessed by visual analogue scale.

### Assessment of study quality

All relevant clinical studies were evaluated for methodological quality using Jadad scale (9) by two reviewers independently. This scale assesses randomization describing (0-2 points), randomization concealment (0-2 points), blinding (0-2 points), and dropouts and withdrawals (0-1 points) of RCTs. Jadad score ≤3 or ≥4 indicates low or high quality respectively. Additionally, guidelines in the Cochrane Handbook for Systematic Reviews of Interventions was also used to assess the quality (10). This assessment tool contains six core items: random sequence generation, allocation concealment, blinding of participants and personnel, blinding of outcome assessment, incomplete outcome data, selective reporting and other bias. Each study was classified as having low, unclear, or high risk of bias. We synthesized qualitative information by using Review Manager (Revman, version 5.3, The Nordic Cochrane Center, Copenhagen, Denmark).

#### Statistical analysis

Statistical analysis was conducted with RevMan v.5.3. The primary endpoint was SFR, the second endpoints were clearance time, incidences of complications. Odds ratio (OR) was used for binary variables, and mean difference was used for the continuous parameters. Pooled estimates were calculated with fixed-effect model (Mantel-Haenszel method) if I2 <50%; otherwise, the random-effect model (DerSimonian-Laird method) was applied. The pooled effects were determined by the z test with p ≤0.05 considered statistically significant. Subgroup analyses were conducted according to stones characteristics and geographic regions. Funnel plots were applied for the assessment of publication bias.

## RESULTS

### Study characteristics

**Supplementary Figure 2:**
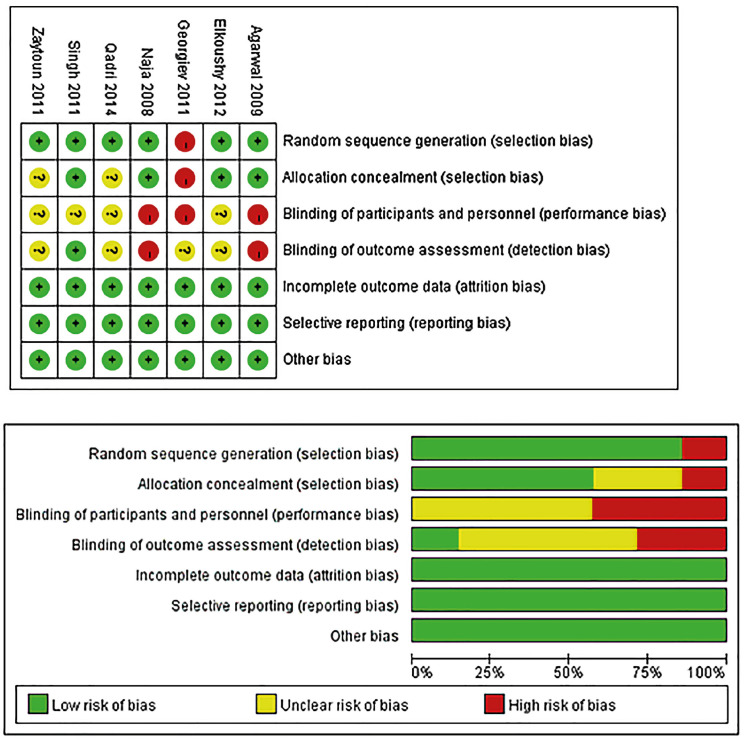
Risk-of-bias analysis: (A) Risk of bias summary: Review authors’ judgments about each risk of bias item for each included study. (B) Risk of bias graph: Review authors’ judgments about each risk of bias item presented as percentages across all included studies.

Through full-text evaluation, 7 studies (11-17) met our inclusion criteria, including 805 patients. Table-1 lists the characteristics of the included studies. According to the Jadad scores, 6 studies were high quality and 1 study was low quality due to inappropriate randomization method. Supplementary Figure-2 shows the details for risk of bias tool.

**Table 1:** Characteristics of included studies.

Author, year	Pts(n)		Ethnicity	Stone location	Stone size, mm	Treatment	SFRs, %	Duration of therapy	Imaging modalities	Standard of repeated ESWL	Standard of stone-free		Jadad score
Naja et al., 2008 (17)		51/65	Asian	Renal	5-20	Tamsulosin 0.4mg/Non-placebo	94.1/75.4	3 months	KUB	Not stated	<3mm		5
Singh et al., 2011 (15)		59/58	Asian	Upper ureteral	6-15	Tamsulosin 0.4mg/Non-placebo	84.7/70.7	3 months	KUB and US	Not stated	<3mm		5
Georgiev et al., 2011 (13)		99/87	European and American	Upper ureteral	5-20	Tamsulosin 0.4mg/Standard medical care	73.4/55.9	30 days	KUB and US	Not stated	<3mm		1
Qadri et al., 2014 (12)	60/60		Asian	Renal	6-20	Tamsulosin 0.4mg/Non-placebo	96.7/80	8 weeks	KUB	Not stated	Not stated		4
Agarwal et al., 2009 (14)	20/20		Asian	Upper ureteral	<15	Tamsulosin 0.4mg/Placebo	95/90	3 months	KUB	Not stated	Not stated		5
Zaytoun et al., 2011 (16)		50/50	European and American	Renal	<20	Tamsulosin 0.4mg+ phloroglucinol / Phloroglucinol	92/84	12 weeks	KUB and US	Not stated	<3mm	4	
Elkoushy, 2012 (11)		63/63	African	Renal, upper ureteral	≤20	Tamsulosin 0.4mg/Non-placebo	87.3/73	3 months	KUB	Not stated	≤3mm	5	

**Pts** = patients; **ESWL** = extracorporeal shock wave lithotripsy; **SFRs** = stone-free rates

### Outcomes

#### SFR

**Figure 1:**
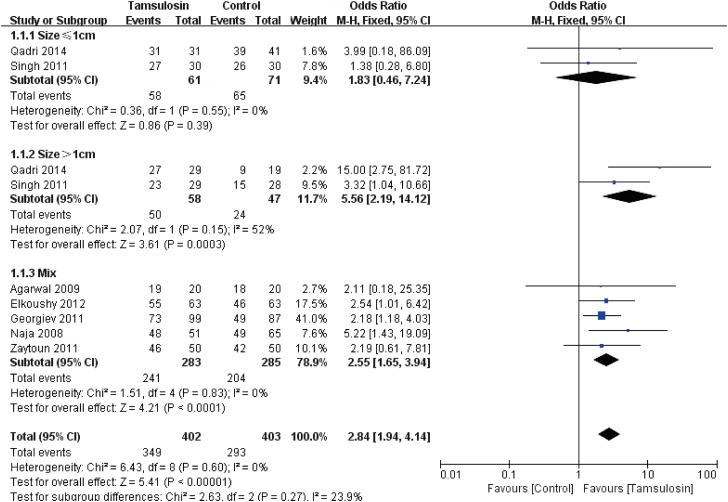
Forest plots with stone clearance as the outcome according to the size.

Tamsulosin provided a higher SFR (see Figure-1) (OR: 2.84; 95% CI, 1.94 to 4.14; p <0.00001). A fixed-effects model was used to calculate the OR and 95% CI.

**Supplementary Figure 3:**
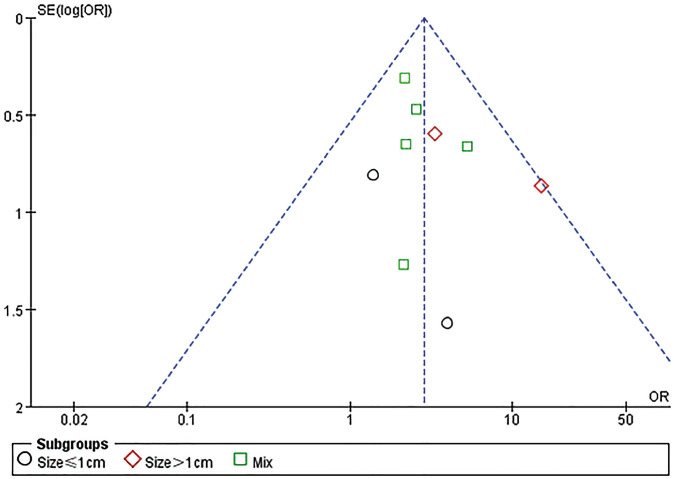
Funnel plot with stone clearance as the outcome according to the size of stone.

A subgroup analysis according to size of stones is also shown in Figure-1. For patients with stones larger than 1cm (OR: 5.56; 95% CI, 2.19 to 14.12; p=0.0003) or mix of large and small stones (OR: 2.55; 95% CI, 1.65 to 3.94; p <0.0001), tamsulosin has significant advantages of SFR over control. However, there was no significant difference of patients with stones less than 1cm between tamsulosin group or control group (P=0.39). No obvious publication bias was found according to funnel plot (see Supplementary Figure-3).

**Figure 2:**
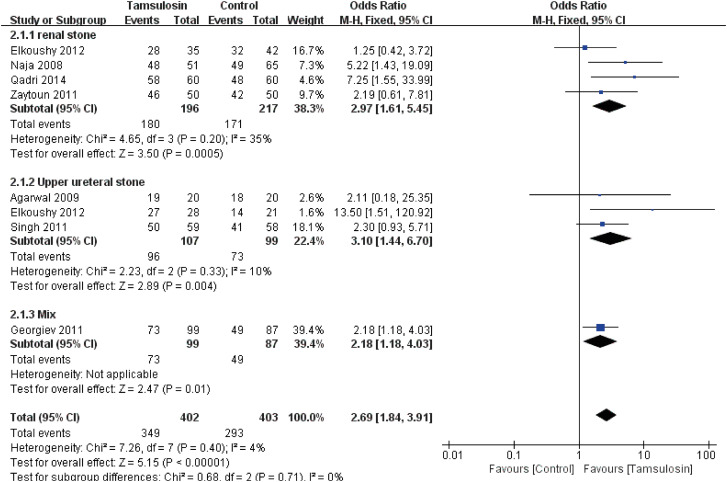
Forest plots with stone clearance as the outcome according to the location of stone.

**Supplementary Figure 4:**
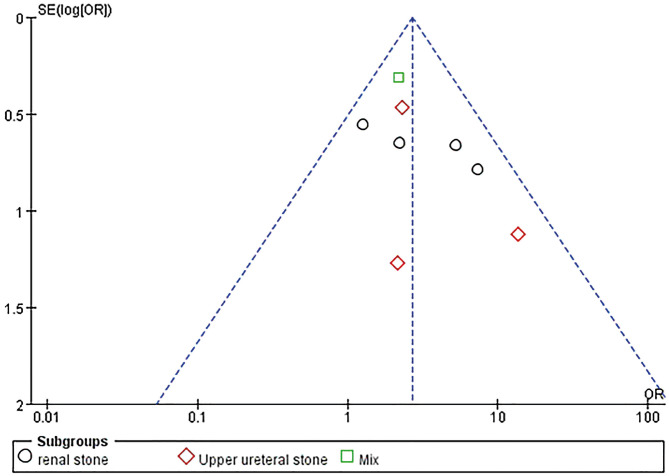
Funnel plot with stone clearance as the outcome according to the location of stone.

A subgroup analysis according to location of stones is shown in Figure-2. For patients with renal stones (OR: 2.97; 95% CI, 1.61 to 5.45; p=0.0005), upper ureteral stones (OR: 3.10; 95% CI, 1.44 to 6.70; p=0.004) or mixed stones (OR: 2.18; 95% CI, 1.18 to 4.03; p=0.01), tamsulosin can provide obvious SFR advantages over control. A fixed-effects model was used to calculate the OR and 95% CI. No obvious publication bias was found according to funnel plot (see Supplementary Figure-4).

**Figure 3:**
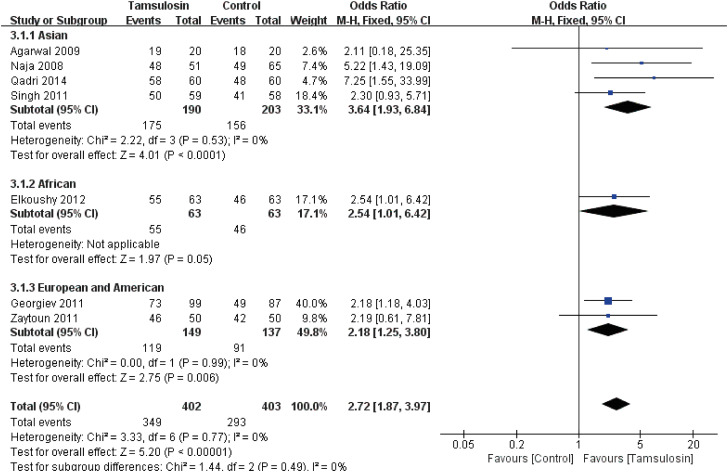
Forest plots with stone clearance as the outcome according to geographic regions.

**Supplementary Figure 5:**
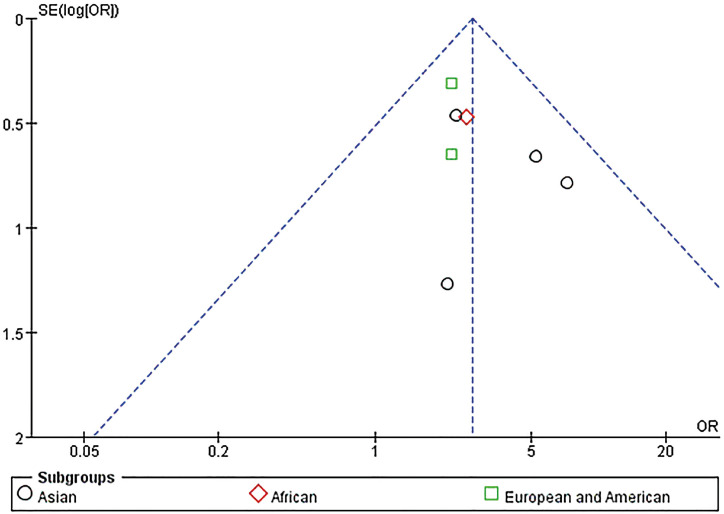
Funnel plot with stone clearance as the outcome according to geographic regions.

A subgroup analysis according to geographic regions is shown in Figure-3. Tamsulosin can provide advantages for patients from any geographic regions including Asian (OR: 3.64; 95% CI, 1.93 to 6.84; p <0.0001), African (OR: 2.54; 95% CI, 1.01 to 6.42; p=0.05) and Euro-American (OR: 2.18; 95% CI, 1.25 to 3.80; p=0.006). A fixed-effects model was used to calculate the OR and 95% CI. No obvious publication bias was found according to funnel plot (see Supplementary Figure-5).

#### Clearance time

**Figure 4:**
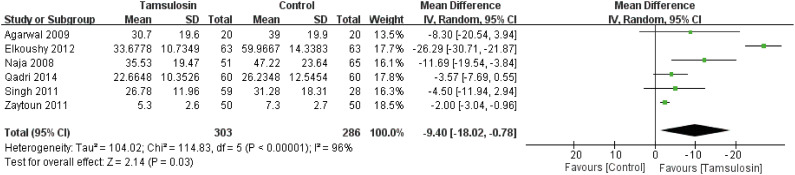
Forest plots with clearance time as the outcome.

The comparison of stone clearance time between tamsulosin and control is shown in Figure-4. Tamsulosin leads to shorter clearance time (WMD: −9.40; 95% CI, −18.02 to 0.78; p=0.03). A random-effect model was used to calculate the WMD and 95% CI.

### Complications

#### Incidences of colic

**Figure 5:**
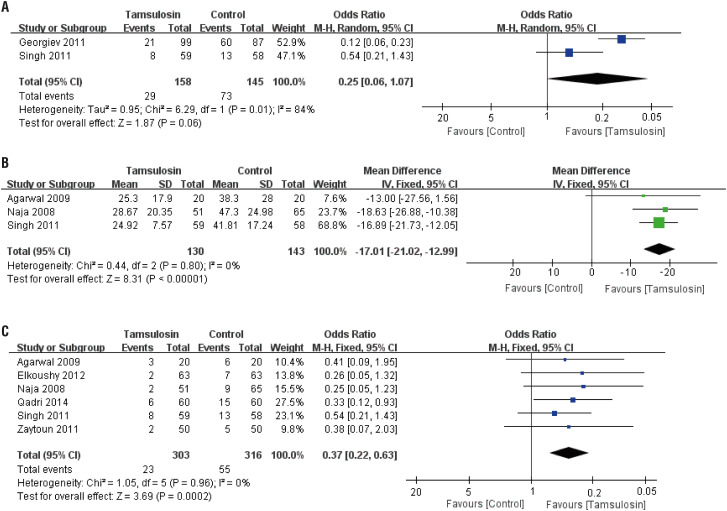
Forest plots with complications as the outcome for: A) incidences of colic; B) pain intensity; C) incidence of steinstrasse. Pain intensity was assessed by visual analogue scale.

The comparison of incidences of colic between tamsulosin and control is shown in Figure-5A. Tamsulosin shows little incidences of colic benefit (OR: 0.25; 95% CI, 0.06 to 1.07; p=0.06). OR and 95% CI were calculated by random-effect model.

#### Pain intensity

The comparison of pain intensity between tamsulosin and control is shown in Figure-5B. Tamsulosin shows significant pain intensity benefit (WMD: −17.01; 95% CI, −21.02 to −12.99; p<0.0001). WMD and 95% CI were calculated by fixed-effect model.

#### Incidence of steinstrasse

The comparison of incidences of steinstrasse between tamsulosin and control is shown in Figure-5C. Tamsulosin shows significant incidence of steinstrasse benefit (OR: 0.37; 95% CI, 0.22 to 0.63; p=0.0002). OR and 95% CI were calculated by fixed-effect model.

#### Adverse reactions

Five studies mentioned the adverse reactions of tamsulosin including variations in blood pressure, headache, dizziness, gastrointestinal problems, or allergic reactions (11, 12-14, 16, 17). The other two studies did not mention adverse reactions (12, 15). Of these five studies, three studies reported the number of patients with those adverse reactions. Two (3.9%) patients in the study reported by Naja et al. (17), 16 (32.0%) patients in the study reported by Zaytoun et al. (16), and 5 (8.0%) patients in the study reported by Elkoushy (11). Tamsulosin was well tolerated by most patients. Among all the 805 included patients, only one patient (a 55-year-old woman) developed symptomatic postural hypotension and required tamsulosin discontinuation (17).

## DISCUSSION

Though ESWL is one of the first-line therapy modalities used for the treatment of urolithiasis, the rate of recurrent therapy remains high. Many patients received ESWL for more than once. After ESWL, the stone clearance rate is dependent on ureteral factors such as ureteral edema and spasm as well as fragment size (18-20). Because tamsulosin can inhibit basal tone and peristaltic ureteral contractions, dilate the ureteral lumen and increase of the fluid bolus volume, it has been used for promoting stone expulsion (21-23). It can also act on the C fibers to block pain conduction (24).

In spite of contradictory results, several RCTs and meta-analyses support MET after ESWL to be used as adjunct to expedite expulsion, increase SFRs and reduce analgesic requirements (4-6, 17, 25-28). For example, a meta-analysis by Chen K et al. also demonstrated that tamsulosin combined with ESWL can provide gratifying achievements for renal, upper ureteral and lower ureteral stones (6). But they did not stratify the results based on different characters of stone or geographic areas. In addition, for patients received repeated ESWL, of whom the size of stone fragments might be smaller, the value of adjunctive MET was not fully assessed. Our systematic review and meta-analysis included several researches and evaluated the efficacy of tamsulosin as an adjunctive therapy for repeated ESWL on different stone sizes, geographic regions, and compared the incidence of steinstrasse and colic, which have not been discussed in the previous meta-analysis.

This study demonstrates a higher profitable effect of tamsulosin on SFR after treatment of repeated ESWL. It has been reported that the size of the stone has a major influence on the success of MET. In our previous multi-cohort RCT study, results suggested that tamsulosin benefits patients with distal ureteral stones by facilitating stone passage and relieving renal colic, and provides a significant expulsion rate for stones >5mm (29). Furthermore, guidelines of European Association of Urology recommend treatment of ARBs as one of MET for distal ureteral stones larger than 5mm (30). Similarly, the size of stones also has prominent effect on success of adjunctive MET. In this study, subgroup analysis based on stone size validated that tamsulosin provide SFR benefits for primary stones larger than 1cm. One possible reason for this difference may be that for stones less than 1cm, the stone fragments produced by ESWL may less than 5mm, which can pass through ureter spontaneously without MET. In our meta-analysis, stones location did not seem to affect the efficacy of adjunctive MET for repeated ESWL, because our pooled data demonstrated that it is equal effective for stones in renal compared with upper ureteral at 1 month treated by tamsulosin after repeated ESWL. Our study shows that SFR is in favor of tamsulosin group, for all different geographic regions. Moreover, our study has also identified a stone clearance time advantage for tamsulosin over control for repeated treatment of ESWL.

As for the complication caused by ESWL, a meta-analysis showed that tamsulosin could reduce the incidence of steinstrasse, colic and pain intensity [6]. Our study also confirmed similar results for repeated ESWL.

Interestingly, two studies demonstrated an insignificant trend in favor of tamsulosin in terms of ESWL sessions, which indicated potential advantages of cost saving associated with repeated ESWL (14, 16). More studies are needed to confirm this advantage.

For the adverse reaction of tamsulosin, no unexpected adverse reactions were reported in all included studies. About 3.9% to 32% patients showed adverse reactions including variations in blood pressure, headache, dizziness, gastrointestinal problems, or allergic reactions. Tamsulosin was well tolerated by most patients, only one patient (0.12%) developed symptomatic postural hypotension and required tamsulosin discontinuation. Thus, it seems safety to receive adjunctive MET by tamsulosin for the promotion of stone fragments clearance for repeated ESWL.

However, there are some limitations. First, the results may be inconsistent as the sample size is limited in most of the included studies. Second, clinical heterogeneity, such as variations in stone characteristics, evaluation of stone removal, types of lithotripsy, and technical details of ESWL, can affect the outcome. Third, in most of the included RCTs, stone status during follow-up was assessed by abdominal simple film instead of computed tomography (CT). However, CT is more sensitive than abdominal simple film (21). And CT is more accurate when used to assess the size of residual stone fragments. Last, none of included studies evaluates efficacy for middle or lower ureteral stones, which may because that ESWL is not suitable for those stones due to bony pelvis and overlying bowel.

Several important steps have been taken to alleviate these limitations. First, we have systematically and comprehensively searched relative RCTs in multiple online databases. Second, the inclusion criteria were rigorously defined, biases from other processing were eliminated, and data were extracted by two independent evaluators. Third, the RCTs with only abstracts of the conference and articles without the full text were excluded to guarantee the quality of this study.

## CONCLUSIONS

In conclusion, adjunctive MET with tamsulosin is effective in patients with specific stone size or location received repeated ESWL. However, no well-designed RCT that used CT for the detection and assessment of residual stone fragments was found. More studies with high quality and the comparison between tamsulosin and secondary ESWL are needed in the future.

## ABBREVIATIONS

ESWL: extracorporeal shockwave lithotripsy
MET: medical expulsive therapy
RCT: randomized controlled trial
ARB: alpha-receptor blocker
SFR: stone-free rate
WMD: weighted mean difference
CI: confidence interval
CT: computed tomography
